# Climate Change and Its Impact on the Eco-Environment of the Three-Rivers Headwater Region on the Tibetan Plateau, China

**DOI:** 10.3390/ijerph121012057

**Published:** 2015-09-25

**Authors:** Chong Jiang, Linbo Zhang

**Affiliations:** 1 College of Global Change and Earth System Science, Beijing Normal University, Beijing 100875, China; 2 State Key Laboratory of Environmental Criteria and Risk Assessment, Chinese Research Academy of Environmental Sciences, Beijing 100012, China; 3 Key Laboratory of Regional Eco-Process and Function Assessment and State Environment Protection, Chinese Academy of Environmental Sciences, Beijing 100012, China; 4 Joint Center for Global Change Studies, Beijing 100875, China

**Keywords:** climate change, water resources, water environment, vegetation growth, soil erosion, glacier, snow

## Abstract

This study analyzes the impact of climate change on the eco-environment of the Three-Rivers Headwater Region (TRHR), Tibetan Plateau, China. Temperature and precipitation experienced sharp increases in this region during the past 57 years. A dramatic increase in winter temperatures contributed to a rise in average annual temperatures. Moreover, annual runoff in the Lancang (LRB) and Yangtze (YARB) river basins showed an increasing trend, compared to a slight decrease in the Yellow River Basin (YRB). Runoff is predominantly influenced by rainfall, which is controlled by several monsoon systems. The water temperature in the YRB and YARB increased significantly from 1958 to 2007 (*p* < 0.001), driven by air temperature changes. Additionally, owing to warming and wetting trends in the TRHR, the net primary productivity (NPP) and normalized difference vegetation index (NDVI) showed significant increasing trends during the past half-century. Furthermore, although an increase in water erosion due to rainfall erosivity was observed, wind speeds declined significantly, causing a decline in wind erosion, as well as the frequency and duration of sandstorms. A clear regional warming trend caused an obvious increasing trend in glacier runoff, with a maximum value observed in the 2000s.

## 1. Introduction

The Intergovernmental Panel on Climate Change (IPCC) Fifth Assessment Report on Climate Change reported that during the past half-century, almost all regions across the globe experienced warming [1], with the fastest warming region shown to be the mid-latitudes of the Northern Hemisphere [2]. The report further showed that global climate change is caused by a combination of both natural and man-made factors. However, human activity is very likely to be the main reason for global warming since the mid-20th century, with a likelihood of more than 95% [1]. Rising temperatures accelerate global water and carbon cycles, exacerbate extreme hydrological and climatic events and lead to worldwide redistributions of water resources and ecosystems at various scales [3]. Moreover, ecological conservation and restoration are necessary to mitigate the effects of environmental degradation, and China has contributed significantly to such actions [4]. Determining exactly how global climate change and ecological conservation affect the eco-environmental balance has been an important research focus for the IPCC, the International Geosphere-Biosphere Program (IGBP), the Future Earth-International Council for Scientific Unions (ICSU) and other international organizations and projects. In China, during the past few decades, the regional climate in the western region has become warmer and wetter [5,6,7], and this trend is likely to continue [3,5]. Owing to the contribution from meltwater, the water system in the arid and semi-arid regions of western China is highly fragile in the context of global and regional warming. In addition to the impact on water resources, climate change can influence other aspects of eco-environments, such as land degradation and soil erosion [8], carbon cycles [9], vegetation growth and productivity [10], glaciers, permafrost and snow cover melt [3,5,11,12,13,14,15].

The Three-Rivers Headwater Region (TRHR) on the Tibetan Plateau, China, is located in the mid-latitudinal regions of Eurasia and is one of the most globally-sensitive areas regarding climate change responses [3,16,17]. Owing to its unique geographical location, rich natural resources and ecological importance, this region serves as a critical natural buffer on the Tibetan Plateau, China [8,9]. However, owing to the high altitude and harsh natural conditions, its eco-environment is highly fragile. Recently, this eco-environment has undergone significant changes owing to the impact of climate change and increased human activities. For example, glacial retreat, rising snow lines, grassland degradation and a decline in water conservation capacity pose direct threats to the ecological balance in the TRHR [8,9]. In view of this background, the state council promulgated and implemented the Overall Planning of Eco-environment Protection and Construction in TRHR, also known as the TRHR Project, in 2005.

Domestic and international researchers have conducted systematic and in-depth studies at both regional and local scales on eco-environmental changes in the TRHR, including studies on grassland degradation [8], vegetation cover change [10], land use and cover change [17], soil organic carbon [18] and streamflow regulating functional change [19,20]. However, this research has focused on eco-environmental changes after the implementation of ecological projects. In other words, although dynamic monitoring and effectiveness assessments of single and multiple ecological projects have been conducted, few analyses exist that have focused on the causes of eco-environmental change and on how results from regional-scale variations might be understood in the context of larger-scale climate change. In addition, most of the research has focused on recent years, with little information available on eco-environmental changes prior to 2005 and few studies encompassing more than 30 years of data. Several important eco-environmental phenomena in the TRHR, such as soil erosion, sandstorm events and water temperature changes, have not been adequately analyzed and understood. Therefore, there is an urgent need for in-depth investigation on eco-environmental fluctuations and responses to climate change, which will play a crucial role in future sustainable development.

Therefore, the objectives of this study are: (1) to quantitatively assess the eco-environmental changes over the past half-century in the TRHR through observations and model simulations of, for example, water resources, the water environment, soil erosion, sandstorm events, vegetation growth and glacier and snow-cover melting; (2) to investigate the influential factors for eco-environmental changes; and (3) to discuss the policy and practical implications of eco-environmental changes and to provide a reference for eco-environmental policy-making and planning. The results of these analyses will allow an assessment of the following factors: (1) the coexistence of positive and negative eco-environmental impacts in the TRHR due to climate change; (2) the eco-environmental changes in the TRHR most relevant to climate fluctuations; and (3) the heterogeneity of large-scale climatic and eco-environmental change.

## 2. Data and Methods

### 2.1. General Description of the Study Area

**Figure 1:**
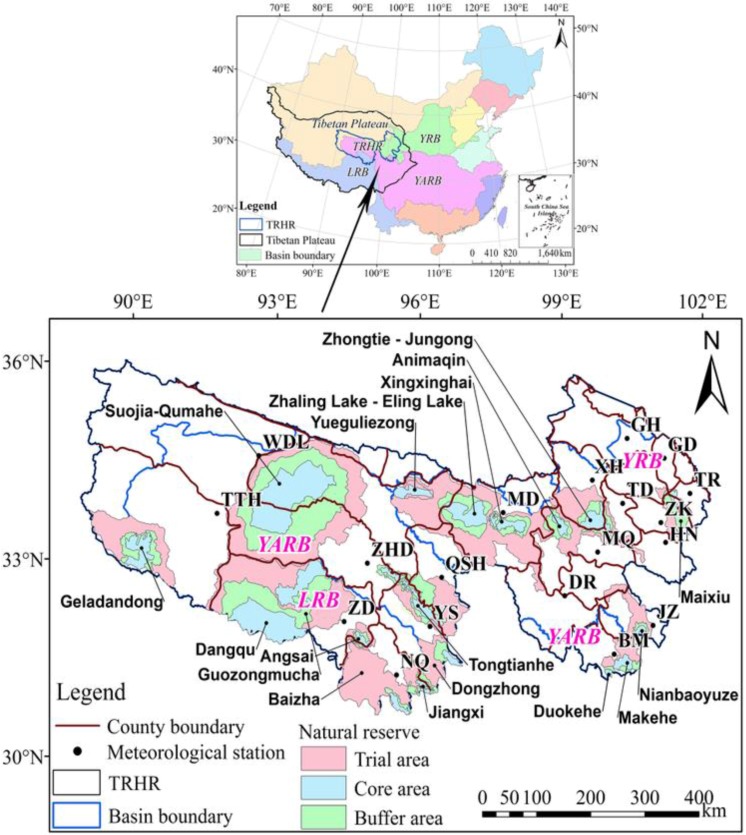
Geographical location, natural conservation area and spatial distribution of meteorological stations in the Three-Rivers Headwater Region (TRHR). YRB, Yellow River Basin; YARB, Yangtze RB; LRB, Lancang RB. WDL, Wudaoliang; TTH, Tuotuohe; ZD, Zaduo; YS, Yushu; NQ, Nangqian; QSH, Qingshuihe; MD, Maduo; DR, Dari; MQ, Maqin; BM, Banma; JZ, Jiuzhi; TD, Tongde; XH, Xinghai; GH, Gonghe; GD, Guide; TR, Tongren; ZK, Zeku; HN, Henan.

The TRHR includes 22 counties and cities, with a total area of 39.5 × 10^4^ km^2^, and includes complex terrain in which mountains form the basic framework of the landscape (Figure 1). The annual average temperature ranges from −5.6 °C to 7.8 °C; the total rainfall is between 262.2 mm and 772.8 mm; and the annual daylight hours are between 2300 h and 2900 h. The many years’ average annual runoff is approximately 500 × 10^8^ m^3^, accounting for 25%, 49% and 15% of that in the Yangtze (YARB), Lancang (LRB) and Yellow (YRB) river basins, respectively. Soil types vary with altitude and consist of alpine cold desert soil, alpine meadow soil, alpine steppe soil, cinnamon soil and forest soil. The alpine meadow soil and meadow soil account for the largest proportion, and the permafrost is highly developed [21]. The TRHR Project was launched in 2005 and includes 18 nature reserves of 39.5 × 10^4^ km^2^ in total. The total project investment was 75.07 billion RMB, including the return of pasture and farmland to forest, afforestation and comprehensive treatment for soil degradation in black soil beaches. The project known as “Returning Pasture and Farmland to Forest” accounted for the largest proportion of finances (41%) from the TRHR Project. Most projects were completed by 2013, as the budget execution rate reached more than 95%. The TRHR project has achieved remarkable success in eco-environmental development [21].

### 2.2. Data

The data sources and detailed information on the datasets used in this research can be found in Table 1. Excluding several sites that have significant quantities of missing data, the data used in this study were collected from 19 ground-based meteorological stations in the TRHR and neighboring regions, operated by the China Meteorological Administration (CMA). The spatial distribution of the meteorological stations can be seen in Figure 1. Complete records for almost all of the climatic factors from 1956 to 2012 were obtained, and cokriging and inverse distance weighted techniques were used to interpolate missing data. The records of sandstorm events during 1954 to 2007 were also collected. Three hydrological sites were chosen within the TRHR and its tributaries, including complete records of streamflow, sediment concentration and water temperature during 1956 to 2012. Landsat Thematic Mapper (TM)/Enhanced Thematic Mapper (ETM) images recorded in 2000 and 2010 were used to extract land cover data for the TRHR. Prior to interpretation, the remote sensing data was geo-referenced using 1:100,000 topographic maps. The land cover types were identified using ArcMap based on the spectral reflectance and structure of objects. The 27 land cover subtypes identified in the study area were further grouped into six aggregated types, including woodland, grassland, farmland, residential areas, water bodies and desert, with a classification accuracy of 95%. The GIMMS NDVI (Global Inventor Modeling and Mapping Studies, Normalized Difference Vegetation Index) data for 1982 to 2006 were used to investigate the vegetation activity changes. To obtain additional time series data, Moderate-Resolution Imaging Spectroradiometer (MODIS) data with a spatial resolution of 0.05° recorded during 2000 to 2012 were collected. In this study, two types of data sources in the overlap period of 2001 to 2006 were collected to maintain the data consistency. The statistical results for the theoretical and actual livestock capacity in the TRHR in 2008 were collected from the Agriculture and Animal Husbandry of Qinghai Province. The rodent area in 2006, based on a field investigation, was collected from grassland monitoring stations in every county included in the study.

**Table 1:** Datasets for climate and eco-environmental change assessments, including data types, dataset names, specific information and sources.

Data Types	Dataset Name and Specific Information	Source
Basic geographic information	The project planning map of second phase of the TRHR Project	Remote Sensing Monitoring Center of Eco-environment in Qinghai Province, China [22]
	Administrative map (scale: 1:4,000,000)	The National Geomatics Center of China [23]
	Digital elevation model (30 m × 30 m)	U.S. Geological Survey [24]
	River and lake system atlas	The National Geomatics Center of China [23]
The social and economic data	Statistical yearbook for 22 counties of TRHR in 2008	Bureau of Statistics in Qinghai Province, China
Hydrological and meteorological data	Monthly streamflow data in 3 national standard hydrological stations during 1956 to 2012 (Tangnaihai Station in YRB, Zhimenda Station in YARB and Xiangda Station in LRB)	Bureau of Hydrology and Water Resources Survey in Qinghai Province, China
	Daily meteorological data in 19 national standard weather stations during 1956 to 2012	National Meteorological Data Sharing Service System [25]
	The records of sandstorm events during 1954 to 2007	
	Monsoon intensity index during 1956 to 2012	National Climate Center [26]
Remote sensing image	MODIS NDVI product during 2000 to 2012 (resolution: 1km×1km)	Cold and Arid Regions Science Data Center [27]
	AVHRR NDVI product during 1982 to 2006 (resolution: 8 km × 8 km)	
	TM and ETM images in 2000 and 2010 (resolution: 30 m × 30 m)	U.S. Geological Survey [24]
Soil data	Soil map (scale: 1:1,000,000)	Institute of Soil Science, Chinese Academy of Sciences

### 2.3. Methods

#### 2.3.1. Framework and Indicators for Eco-Environmental Change Assessments

To quantitatively assess eco-environmental change in the context of climate change, we established a framework, and several indicators were chosen based on actual observations and model simulations. The eco-environmental changes include water resources, water environment, soil erosion, vegetation growth and glacier and snow-cover melting; the specific indicators and assessment methods can be found in Table 2.

**Table 2:** Framework and indicators for eco-environmental change assessments. NPP, net primary productivity.

Assessment Projects	Indicators	Unit	Assessment Method
Water resources	Annual and seasonal flow	m^3^/s	Observational record
	The ratio of flow in dry season to annual flow	%	Observational record
Water environment	Sediment concentration	kg/m^3^	Observational record
	Water temperature	°C	Observational record
Vegetation growth	NPP	kg·C·hm^–2^·a^–1^	Thornthwaite–Memorial model
	NDVI	Dimensionless	GIMMS NDVI and MODIS NDVI
Soil erosion	Water erosion amount	t	USLE model
	Rainfall erosivity	MJ·mm·hm^–2^·h^–1^·a^–1^	Half-month rainfall erosivity model
	Wind erosion amount	t	RSWQ model
	Frequency and duration of sandstorm event	Times or minute	Observational record
Snow cover and glaciers melting	Freezing layer height	m	Section 2.3.4
	>0 °C annual cumulative temperature	°C	Observational record
	Glacier runoff depth	mm	Modified degree-day model

Note: ‘hm^2^’ represents hectare, and ‘a’ is same as year (yr).

#### 2.3.2. Trend Analysis with the Mann–Kendall Non-Parametric Test

In this study, we used the Mann–Kendall test to detect trends in related meteorological and hydrological factors. This test is often used to detect significant trends in time series data [28].

#### 2.3.3. Double Mass Curve

The double mass curve is a simple and practical observation method widely used in the study of long-term trends in hydro-meteorological data [29]. This method was first used by Merriam to examine the consistency of precipitation data in the Susquehanna watershed in the United States [30], and Searcy later reported a theoretical explanation of this technique [31]. The theory of the double-mass curve is based on the fact that a plot of two cumulative quantities over the same period should show a linear relationship provided the proportionality between the two remains unchanged and the slope of the trend line is proportional. As a result, this method can be used to smooth a time series and to suppress random elements, and it can therefore be used to show the main trends in a time series. In this study, double-mass curves of sediment concentration *versus* streamflow, as well as streamflow *versus* rainfall, were plotted. The appearance of an inflection point in these plots indicates the point at which the relationship between the two parameters begins to change significantly [29].

#### 2.3.4. Estimation of the 0 °C (Freezing) Layer Height

A linear interpolation method was used to calculate the freezing layer height (FLH) at each time, assuming that the temperatures between the two standard barospheres of 500 hPa and 850 hPa changed uniformly in the vertical direction. Subsequently, the mean of two times (8:00 a.m. and 8:00 p.m.) was calculated to obtain the daily FLH. Finally, daily values from June to August were averaged to obtain the summer FLH. A linear interpolation method was used to calculate the FLH with the following Equation:

(1) $$H_{i}=\frac{H_{j}-H_{k}}{T_{j}-T_{k}}\left(T_{i}-T_{k}\right)+H_{k},$$

where *H* represents height (m), *T* represents temperature (°C), *i* identifies the FL and *j* and *k* identify the upper and lower standard barospheres of the FL, respectively.

#### 2.3.5. Potential Evapotranspiration (*ET*_0_) Estimation with the Penman–Monteith Equation

Following the recommendation of the Food and Agriculture Organization (FAO), the Penman–Monteith equation was modified to better account for specific local conditions [32]. In this study, several parameters were calibrated using the observed data for each station in the various regions for the estimation of net radiation (*R*_n_). This research adopts the Penman–Monteith method to estimate the daily *ET*_0_ (mm·d^−1^):

(2) $$ET_{0}=\frac{0.408\Delta (R_{n}-G)+r\frac{900}{T+273}U_{2}(e_{s}-e_{a})}{\Delta +r(1+0.34U_{2})},$$

where *ET*_0_ is potential evapotranspiration, *R_n_* is the net radiation at the crop surface (MJ ·m^−2^·d^−1^), MJ represents mega joule, *G* is the soil heat flux density (MJ ·m^−2^·d^−1^), *T* is the air temperature at a height of 2 m (°C), *U*_2_ is the wind speed at a height of 2 m (m·s^−1^), *e*_s_ is the saturation vapor pressure (kPa), *e*_a_ is the actual vapor pressure (kPa), Δ is the slope of the vapor pressure curve (kPa·°C^−1^) and *γ* is the psychrometric constant (kPa·°C^−1^).

#### 2.3.6. Climate Productivity Estimation with the Thornthwaite–Memorial Equation

This research used the concise and practical Thornthwaite–Memorial model, which was developed by Lieth [33], to estimate climate productivity. By considering the light, temperature and water conditions on dry matter generation, this model effectively characterized the relationship between climate productivity and photosynthesis to clearly demonstrate the impact of climate change on productivity:

(3) $$P_{v}=3000\left(1-e^{-0.000956\left(v-20\right)}\right)$$

(4) $$v=1.05R/{\left[1+{\left(1.05R/L\right)}^{2}\right]}^{1/2}$$

(5) $$L=300+25t+0.05t^{3}$$

where *P_v_* is climate productivity (kg·hm^−2^·a^−1^), *v* is annual actual evaporation (mm), *R* is annual rainfall (mm), *L* is annual maximum evaporation (mm) and *t* is annual mean temperature (°C).

#### 2.3.7. Glacier Mass Balance and Glacier Runoff Simulations Using a Modified Degree-Day Model

Glacier mass balance and glacier runoff simulations were conducted in monthly time steps using a degree-day model based on the principals of the original degree-day model, modified to monthly time steps. This degree-day model is based on an assumed relationship between ablation and air temperature, usually expressed in the form of positive temperature sums [34,35]:

(6) *A* = *DDF* × *PDD*

where *DDF* is the degree-day factor, which is different for snow and ice surfaces (mm·d^−1^·°C ^−1^), *A* is the depth of meltwater (mm) and *PDD* is the monthly positive accumulated air temperature, which is given by [36]:

(7) $$PDD=\sum_{i=1}^{n}H_{t}\cdot T_{t}$$

where *T_t_* is the monthly mean air temperature and *H_t_* is a logical variable, which can be defined such that *H_t_* = 1 for *T_t_* ≥ 0 °C and *H_t_* = 0 for *T_t_* < 0 °C. In our study, *PDD* was calculated based on a function derived from the relationship between *PDD* and the monthly average air temperature in addition to the standard deviation of the observations [37,38].

The mean annual mass balance was calculated based on the simulated snow accumulation and the simulated snow and ice melt as follows:

(8) *B_n_* = *P* − *A*

where *B_n_* is the annual mass balance (mm) and *P* is the annual snow accumulation (mm). We used a balance year running from 1 October to 30 September the following year.

For the entire basin, the glacier runoff *Q* for a given year is computed as:

(9) $$Q=\sum_{i=1}^{n}s\left(i\right)\left[\left(1-f\right)A\left(i\right)+P_{liq}\left(i\right)\right]$$

where *s*(*i*) is the glacier area at the *i*-th elevation band derived from the digital elevation model (DEM) and the digital vector of the glaciers, *f* is the refreezing rate and *P_liq_*(*i*) is the liquid precipitation directly transformed to glacier runoff. Additional details on the calculation steps for the monthly degree-day model are reported in [39].

#### 2.3.8. Water and Wind Erosion Assessment

We calculated the soil loss in a land parcel using the universal soil loss equation (USLE) and the revised wind erosion equation (RWEQ). The data input includes the geomorphology, climate, vegetation and management practices. The USLE and RWEQ are the most widely-used methods for soil erosion modelling and assessment and were applied to quantify the annual soil loss. The soil erosion amount calculated by the USLE can be mathematically expressed as:

(10) *E* = *R* × *K* × *L* × *S* × *C_v_* × *P_v_*,

where *E* is the amount of soil erosion (t·ha^−1^·yr^−1^); *R*, *K*, *L* and *S* represent the rainfall erosivity (MJ·mm·ha^−1^·h^−1^·yr^−1^), soil erodibility (t·ha·h·ha^−1^·MJ^−1^·mm^−1^), slope length and slope angle factors, respectively; and *C_v_* and *P_v_* refer to the current vegetation cover and erosion control factors, respectively. *L*, *S*, *C_v_* and *P_v_* are dimensionless factors.

For some key parameters, the soil erosive factor *K* was calculated using the erosion/productivity impact calculator (EPIC) model based on the soil texture [40]. The principle behind this model states that the rainfall erosivity factor *R* equals the kinetic energy of rainfall *E* times the maximum rain intensity observed in a 30-min period (cm·h^−1^) *I*_30_, which requires unavailable data. Therefore, we customized the calculation of the *R* factor for a Chinese context by using the half-month precipitation erosivity [41], which is a parameter embedded in the Chinese soil loss equation [42].

To include full consideration of factors, such as climate, vegetation, surface roughness, soil erodibility and soil crust, the RWEQ model was used to evaluate the amount of wind erosion [43]:

(11) *Q* = 109.8 × (*W* × *K* × *S* × *R* × *C*),

where *Q* is the transport capacity of sediment (kg·m^−1^) per unit length, *W* is the climate factor (m·s^−1^), *K* is the soil erodibility component, *S* is the soil crust factor, *R* is the soil roughness factor and *C* is the vegetation factor, including green and withered vegetation. *K*, *S*, *R* and *C* are dimensionless factors.

## 3. Climate Change in the TRHR

### 3.1. Sharp Temperature and Precipitation Increases in the TRHR

#### 3.1.1. Average Temperature Increase above the Global Average

**Figure 2:**
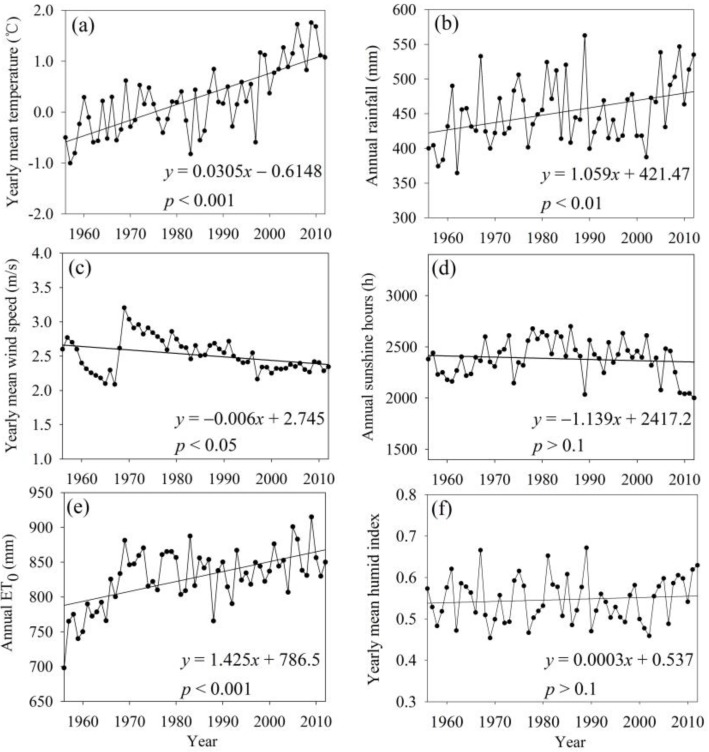
Temporal variations in meteorological factors in the TRHR during 1956 to 2012 including (**a**) precipitation, (**b**) temperature, (**c**) wind speed, (**d**) sunshine hours, (**e**) potential evapotranspiration and (**f**) humidity index.

During the past half century, the maximum rate of temperature increase in the TRHR was 0.31 °C/10 a (Figure 2a), which is significantly higher than the global average of 0.12 °C/10 a [1]. Our analysis showed that a significant temperature rise occurred during the early 1990s; during 1956 to 1990, the average temperature increased to a lesser extent. In 1990, the average annual temperature showed an abrupt change, and the elevated rate of 0.68 °C/10 a indicated an accelerating trend. A statistical analysis of 19 sites in the TRHR demonstrated that in the 1990s, 92% of the stations observed an increasing temperature trend. In the 21st century, 100% of the stations showed an increasing temperature trend with individual temperatures higher than in the 1990s.

Increases in winter temperatures occurred in the TRHR during the past 57 years at a rate of 0.53 °C/10 a. Specifically, the differences in seasonal temperatures showed that winter average temperatures contributed 35.10% to the annual temperature increase rate; therefore, substantially higher winter temperatures may be an important factor in the increased average annual temperature.

#### 3.1.2. Dynamic Increase in Precipitation

During the past half century, precipitation in the TRHR exhibited increases similar to those observed for temperature (Figure 2b). During 1956 to 1990, precipitation levels remained relatively stable, although precipitation showed a sharp increase in 1990. Approximately 80% of the stations showed increasing trends in 1970, followed by a decrease of 75% in the 1980s and an increase of 82% in the 1990s for the entire TRHR. 2000 to 2012 was the most humid period of the past half century. It is worth mentioning that during the last 12 years, the increases in precipitation were enhanced; about 95% of the stations showed higher increases than those recorded in the 1990s.

### 3.2. Other Meteorological Factors

The yearly mean wind speed and annual sunshine hours presented slightly decreasing trends, particularly in the 21st century (Figure 2c,d). The largest negative anomaly occurred during 2000 to 2012 (Table 3). *ET*_0_ presented an increasing trend similar to that observed for temperature (Figure 2e). The largest positive anomaly was seen in the 21st century, followed by that in the 1970s (Table 1). The humidity index (Figure 2f) remained stable before 2000, then sharply increased at a rate of 0.11/10 a.

**Table 3:** Decadal average anomalies for meteorological factors.

Decade	Temperature (°C)	Rainfall (mm)	Wind speed (m/s)	Sunshine hours (h)	*ET*_0_ (mm)	Humidity Index
1960s	−0.40	−10.82	−0.15	−66.53	−28.98	0.004
1970s	−0.23	−3.31	0.32	62.24	18.26	−0.02
1980s	−0.21	23.28	0.09	110.36	4.80	0.02
1990s	0.09	−15.22	−0.08	58.89	3.52	−0.02
2000–2012	0.87	23.98	−0.19	−108.69	27.51	0.01

## 4. The Eco-Environmental Impact of Climate Change

### 4.1. Hydrological Impact

#### 4.1.1. Interannual Variability of Streamflow

**Figure 3:**
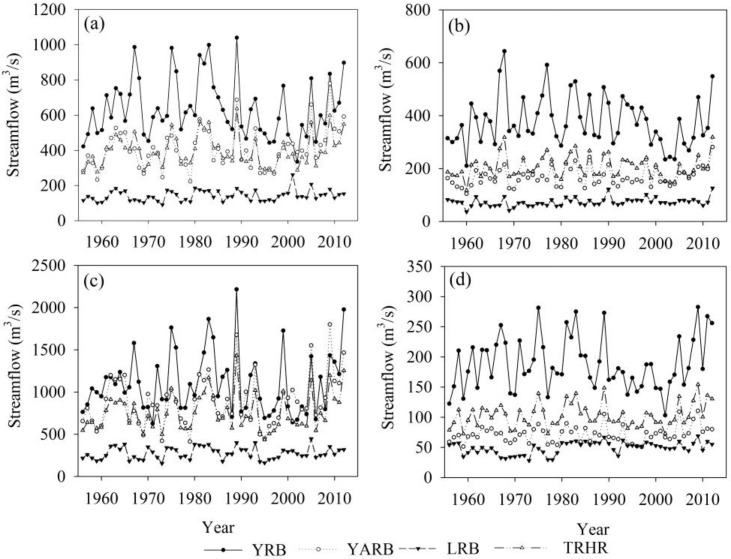
Interannual variations of streamflow (**a**) on an annual scale, (**b**) in the spring and (**c**) summer flood seasons and (**d**) in the dry season in the TRHR from 1956 to 2012.

The TRHR streamflow showed an increasing trend during 1956 to 2012 (Figure 3a), particularly within the past 10 years. Since the implementation of the TRHR project (2005 to 2012), the annual mean streamflow values in the YARB, YRB and LRB were 170.03 × 10^8^ m^3^, 215.2 × 10^8^ m^3^ and 47.73 × 10^8^ m^3^, respectively, 39.3%, 5.2% and 9.7% higher, respectively, than the mean levels between 1956 and 2012. The flow during the spring and summer flood seasons also increased significantly, which is consistent with the annual streamflow data (Figure 3b,c). During the dry season, increases in the ratio of streamflow to annual flow in the YARB, YRB and LRB were, respectively, 1.1%, 1.0% and 1.1% higher than the long-term mean levels, indicating that the streamflow regulation function of the ecosystem was enhanced.

#### 4.1.2. Consistent Relationship for Runoff *vs*. Rainfall and Runoff *vs*. Monsoon

**Figure 4:**
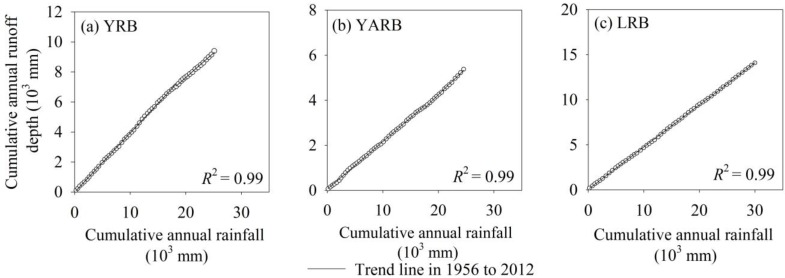
Double mass curves for cumulative rainfall and cumulative runoff depth at the (**a**) Tangnaihai, (**b**) Zhimenda and (**c**) Xiangda stations of the Yellow (YRB), Yangtze (YARB) and Lancang (LRB) river basins, respectively.

The relationship between cumulative annual rainfall and runoff depth (Figure 4) demonstrated that in all three basins (LRB, YRB and YARB), rainfall and runoff were highly uniform during 1956 to 2012, with no change-point detected to reflect the effect of evaporation and anthropogenic activity on runoff. Runoff was mainly influenced by natural rainfall.

**Figure 5:**
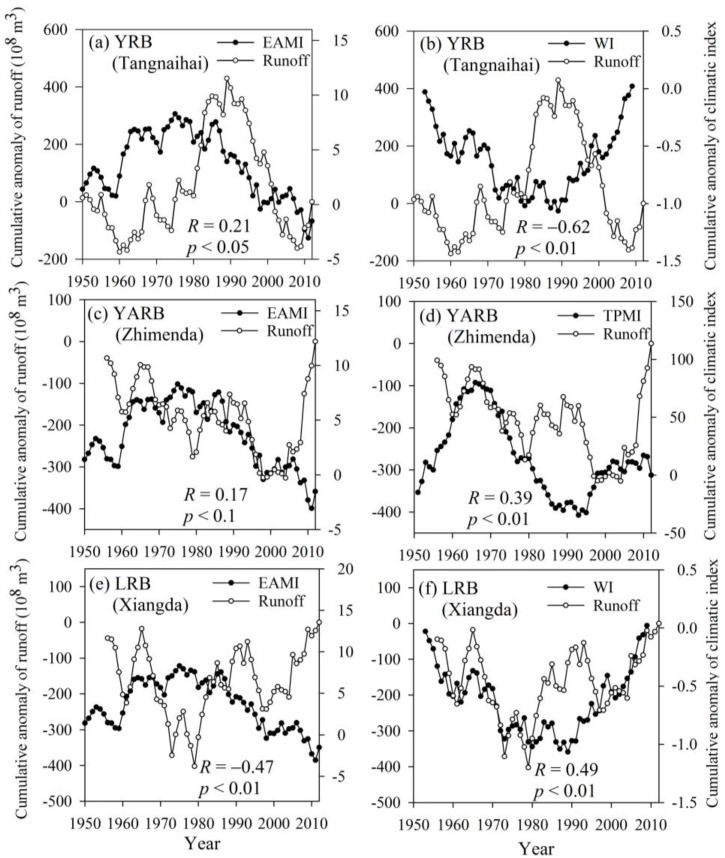
Cumulative anomaly curves of runoff *vs*. the monsoon intensity index in the TRHR. (**a**) and (**b**) are for the YRB, (**c**) and (**d**) are for the YARB, (**e**) and (**f**) are for the LRB. EAMI, WI and TPMI represent the East Asian monsoon index, the westerly index and the Tibetan Plateau monsoon index, respectively. R and *p* represent the correlation coefficient and significance level, respectively.

The ebb and flow of the monsoons determines the start and end of the rainy season, and the strength of the monsoon affects the amount of rainfall. The TRHR is located in the hinterland of the Tibetan Plateau and is directly affected by the monsoon on the Tibetan Plateau itself. However, as the area is located in a zone between prevailing westerlies and the East Asian summer monsoon, runoff in the region is influenced by the combined action of a number of monsoons. Cumulative anomaly curves of runoff *vs*. the monsoon intensity index were drawn to explore this relationship (Figure 5). Runoff in the YRB (Tangnaihai) correlated positively with the East Asian monsoon index (EAMI) and negatively with the westerly index (WI). In years with a strong East Asian monsoon, runoff was high, but in years with strong westerlies, runoff was very low. It is worth noting that the correlation coefficient between runoff and the EAMI was low. We suggest that this is because runoff is affected by a variety of monsoon systems and so lacks direct correspondence to a specific index. In the YARB, the Tibetan Plateau monsoon and the East Asian monsoon play leading roles, resulting in correspondingly high correlation coefficients. This is particularly relevant for the Tibetan Plateau monsoon index (TPMI), which has a correlation coefficient with runoff of 0.39 (*p* < 0.01). The situation in the LRB was similar to that in the YRB, in that runoff was jointly controlled by the East Asian monsoon and westerlies; however, runoff in the LRB gave a higher correlation coefficient than in the YRB.

### 4.2. Water Environment Impact

#### 4.2.1. Sediment Concentration Changes

**Figure 6:**
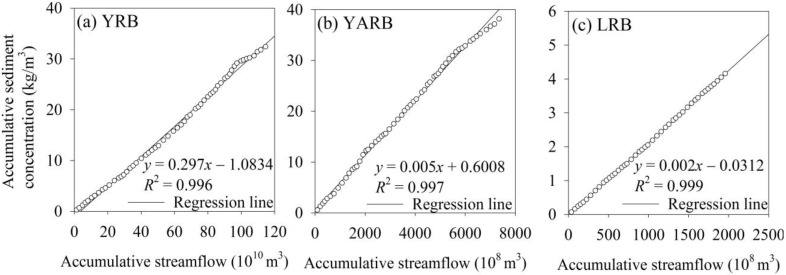
Double mass curve plots of cumulative sediment concentration and cumulative streamflow at the (**a**) Tangnaihai, (**b**) Zhimenda and (**c**) Xiangda stations of the Yellow (YRB), Yangtze (YARB) and Lancang (LRB) river basins, respectively.

To quantify the relationship between sediment concentration and streamflow, a double mass curve was plotted. Figure 6 shows that the streamflow and sediment concentration for the three stations in the TRHR were closely correlated. The double mass curves were almost straight lines, particularly in the LRB and YARB at Xiangda and Zhimenda stations, respectively. Generally, when human activities significantly affect the surfaces that provide the sediment yield, the curve shows an inflection point. Therefore, the linear relationship between sediment and streamflow shown in the figure indicates that human activities had a negligible effect in the TRHR; the sediment concentration observed at hydrological stations was essentially caused by natural precipitation and streamflow.

#### 4.2.2. Water Temperature Change

**Figure 7:**
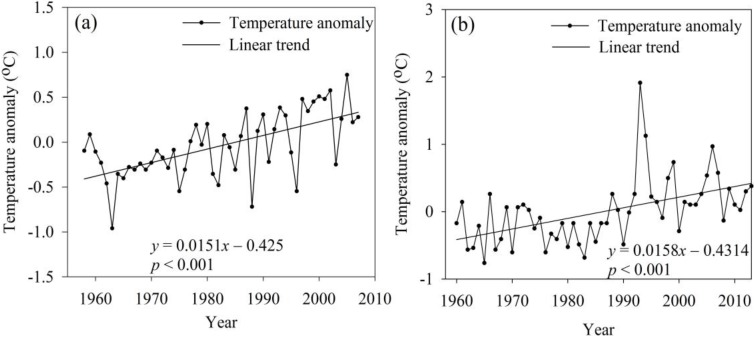
Temporal variations in water temperature anomalies at (**a**) Tangnaihai and (**b**) Batang stations in the Yellow (YRB) and Yangtze (YARB) river basins.

From 1958 to 2007, the water temperature anomaly in the Tangnaihai and Batang stations of the YRB and YARB increased significantly at rates of 0.15 °C/10 a (*p* < 0.001) and 0.16 °C/10 a (*p* < 0.001), respectively (Figure 7). The anomaly at Tangnaihai station varied between −0.96 °C in 1963 and 0.75 °C in 2005, with a range of about 1.71 °C. The anomaly at Batang station varied between −0.76 °C in 1965 and 1.91 °C in 1993, with a range of about 2.67 °C. The water temperature correlated positively with air temperature at these two stations, with respective correlation coefficients of 0.85 and 0.87. Solar radiation provides the heat source for first air and then water; therefore, the water temperature change lags behind that of air on a daily scale. On an annual scale, however, water and air temperature changes are consistent. Owing to the regional warming trend over the last half-century, the water temperature also exhibited a warming trend.

### 4.3. Response of Vegetation Growth to Climate Change

**Figure 8:**
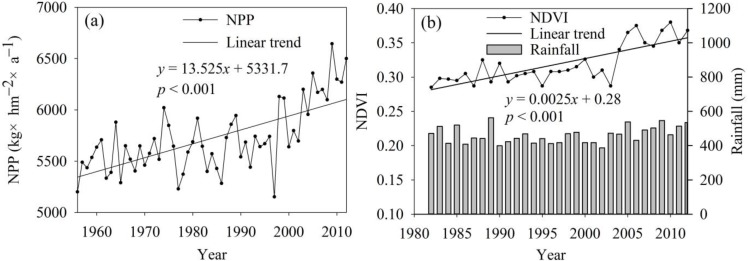
Temporal variations in (**a**) net primary productivity (NPP) from 1956 to 2012 and (**b**) the normalized difference vegetation index (NDVI) from 1982 to 2012 in the TRHR.

Owing to the TRHR warming and wetting trends, which provide sufficient water and heat for vegetation growth, the net primary productivity (NPP) showed a significant increasing trend during 1956 to 2012, with a slope of 13.53 kg C/(hm^2^ a) (*p* < 0.001) (Figure 8a). Regarding the spatial distribution, the NPP increased from southeastern to northwestern areas of the TRHR, with NPPs in the southern YAR and LRB obviously higher than those in the northwestern YARB. From 1956 to 2012, the regions with the most rapid increases in NPP were the southern YRB and LRB. The NDVI in 1982 to 2012, derived from GIMMS and MODIS products, also presented a significant increasing trend with a slope of 0.0025/a (*p* < 0.001) and was closely correlated with NPP and rainfall, as shown in Figure 8b (the correlation coefficient is 0.36 (*p* < 0.01)).

### 4.4. Impact of Climate Change on Soil Erosion

#### 4.4.1. Changes in Rainfall Erosivity and Water Erosion Amounts

**Figure 9:**
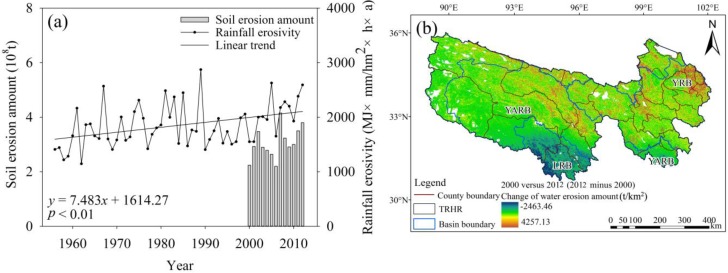
(**a**) Temporal variations in rainfall erosivity during 1956 to 2012 and in water erosion amounts during 2000 to 2012; (**b**) spatial changes in water erosion amounts in the TRHR between 2000 and 2012.

From 1956 to 2012, the rainfall erosivity in the TRHR presented a significant increasing trend with a slope of 7.48 MJ mm/(hm^2^ h a) (*p* < 0.01), which closely matches the rainfall variability (Figure 9a). The water erosion amount calculated using the USLE model showed similar changes to those observed for rainfall erosivity from 2000 to 2012. A comparison of the water erosion amount in 2000 with that in 2012 revealed a slight increase; however, the erosion per unit area clearly increased from 500 to 2500 t/km^2^ (Figure 9b). Within the various basins (Figure 9b), the erosion amounts in the YRB and the northwest inland river basin clearly increased, as did the amounts in several individual YARB sections; however, the amounts in other sections decreased, as did the LRB erosion intensity.

#### 4.4.2. Changes in Wind Erosion Amounts and Sandstorm Event Frequency

**Figure 10:**
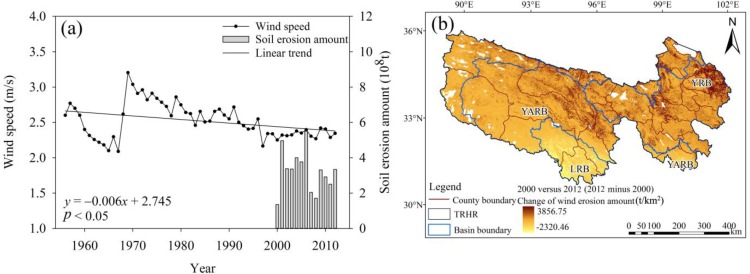
(**a**) Temporal variations in wind speeds during 1956 to 2012 and in wind erosion amounts during 2000 to 2012; (**b**) spatial changes in wind erosion amounts in the TRHR between 2000 and 2012.

Wind is the main driving force for wind erosion. Wind speeds during 1956 to 2012 presented a significant declining trend with a slope of −0.06 m/(s a) (*p* < 0.05), which reduced the erosion force in the same period. Therefore, the wind erosion amounts from 2000 to 2012 presented a trend matching the wind speed trend (Figure 10a). A comparison of the wind erosion amount in 2000 to that in 2012 revealed a slight decrease for most areas of the TRHR (Figure 10b). In the various basins (Figure 10b), the erosion amount in the northeastern YRB increased and the erosion intensity of the LRB was reduced. Some individual sections of the YARB showed increases in the erosion amounts, although others showed decreases.

**Figure 11:**
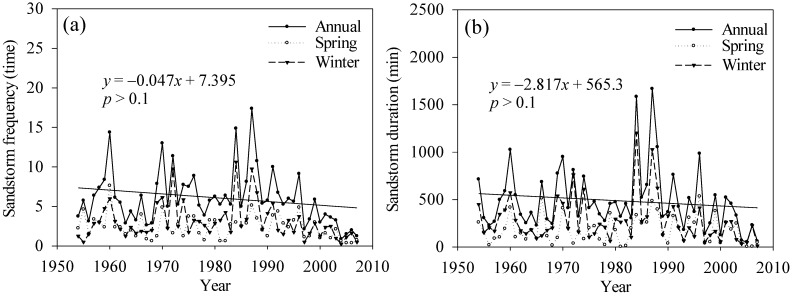
Temporal variations in (**a**) sandstorm frequency and (**b**) sandstorm duration during 1956 to 2012.

The annual and seasonal mean wind speed in the TRHR showed a significant downward trend (Figure 2c). The annual average wind speed declined at the rate of −0.06 m/s·10a (*p* < 0.05), and the wind speed declined in winter and spring at the faster rate of −0.07 m/s·10a (*p* < 0.05). Wind was the main external force behind wind erosion and was generally accompanied by dust events. The number and duration of dust events declined during 1954 to 2007, particularly after the mid-1980s (Figure 11a,b).

### 4.5. Impact of Climate Change on Snow Cover and Glaciers

**Figure 12:**
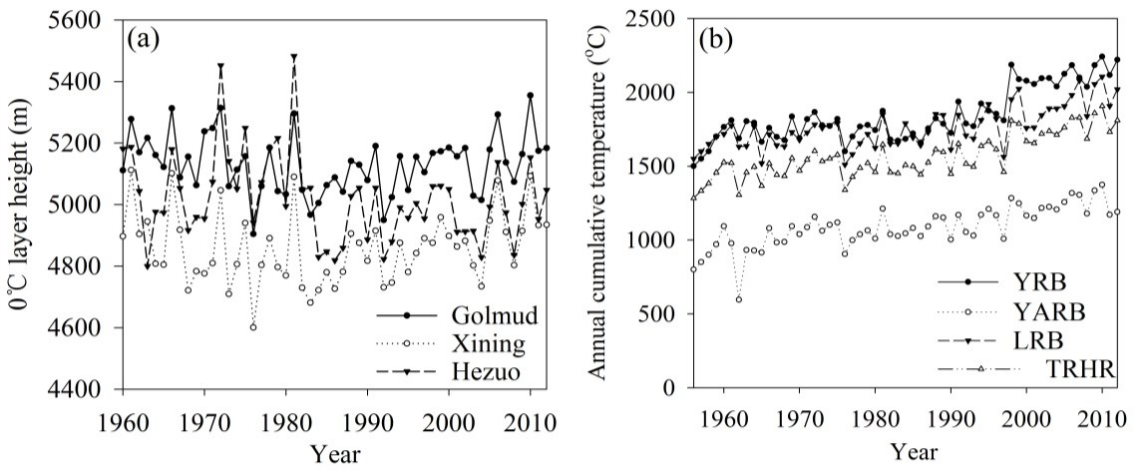
The (**a**) freezing layer height (FLH) and (**b**) >0 °C annual cumulative temperature change in the TRHR during 1956 to 2012.

Because observational data on glaciers and snow cover in the TRHR is generally lacking, this research used the >0 °C annual cumulative temperature and summer freezing layer height (FLH) to reflect the climatic environment under which glaciers and snow cover melt. The FLH is closely related to the snow line [44,45,46,47], and the >0 °C annual cumulative temperature reflects the temperature conditions required for snow cover melting. It should be noted that no sounding stations are located within the TRHR; therefore, stations in the surrounding area were used for this analysis (Figure 11). Figure 12a shows that the FLH increased from 4681.1 m in 1983 to 5153.1 m in 2011, with an average rate of increase of 16.28 m/a (*p* < 0.001). Figure 12b illustrates that the >0 °C cumulative temperature in each basin exhibited significant increasing trends (*p* < 0.001) in the following order: YRB (9.22 °C/a) > TRHR (7.30 °C/a) > YARB (6.62 °C/a) > LRB (6.06 °C/a).

**Figure 13:**
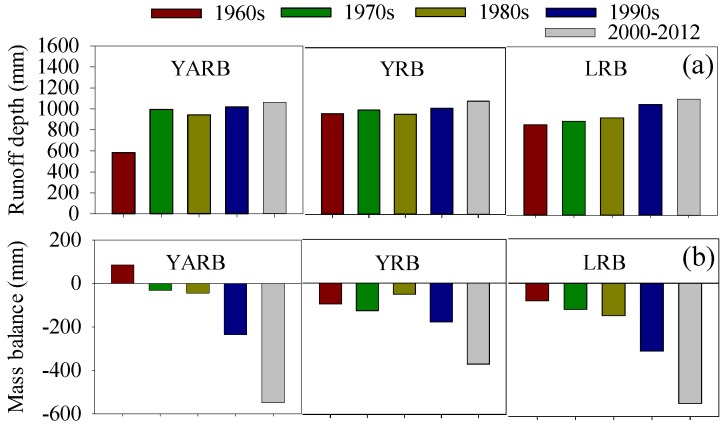
Changes in decadal mean (**a**) glacier runoff depth and (**b**) glacier mass balance in the TRHR during 1960 to 2012.

With the intensification of global warming, glacier recession and increases in the snow line elevation have affected runoff [44,45]; the rate of glacier recession during the last 30 years is 10-times greater than that recorded during the past 300 years [3,11]. The degree-day model estimated the glacier runoff change during 1961 to 2012, as shown in Figure 13a. The glacier runoff presented an obvious increasing trend, with the highest value appearing in the 2000s. The glacier mass balance change, shown in Figure 13b, indicates increasingly large losses since the 1960s. Particularly, during 2000 to 2012, the mass loss was about 549.0 mm in the YARB, 370.5 mm in the YRB and 540.8 mm in the LRB. The differences in the responses of glacier melting to climate change are attributed predominantly to glacier size and the distribution of climate conditions. Furthermore, under the conditions of rapid regional warming, a huge amount of water is released by melting glaciers, as shown in Table 4. The contribution rates of glacier runoff to total runoff, during 1960 to 2012, were 6.7% in the LRB, 1.6% in the YRB and 11.7% in YARB. Similar results have been reported in previous research [45,46,47].

**Table 4:** Estimated glacier runoff since 1960 in the TRHR and its contribution to total runoff.

River System	Glacier Area (km^2^)	Total Runoff (10^8^ m^3^)	Glacier Runoff (10^8^ m^3^)	GR/TR (%)	GR/TR (%) [46]	GR/TR (%) [45]	GR/TR (%) [47]
LRB	316.32	110.5	7.4	6.7	5.4	6.6	4.0
YRB	172.41	245.0	3.9	1.6	1.9	1.3	0.8
YARB	1895.00	215.3	25.2	11.7	18.8	18.5	8.8
GR/TR is the ratio of glacier runoff to total runoff.							

## 5. Discussion

### 5.1. Insufficient Explanation for the Plateau Climate Change Mechanism

Climate change in the TRHR on the Tibetan Plateau is certainly complex. It is difficult to accurately reveal the processes behind the changes and trends from short-term individual results alone. Many domestic and international studies have focused mainly on the analysis of climate characteristics over various time scales at a regional scale [3,5,11,48]. However, such studies are mostly qualitative and lack quantitative data. In particular, none elucidate historical centennial-scale climate variations or the manner in which the mechanisms of climate change affect the water cycle and eco-environment. Specifically, few emphasize the required improvements in understanding the evolution and interaction of climate systems at different temporal scales.

### 5.2. Non-Climate Factors Driving Eco-Environmental Change Acceleration

**Figure 14:**
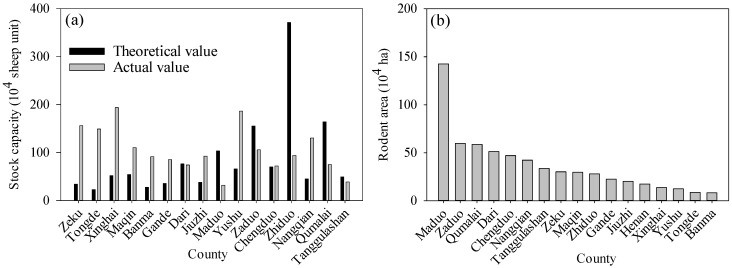
Statistical results for (**a**) livestock capacity in 2008 and (**b**) rodent area in 2006 in the TRHR.

In addition to climate change, non-climate factors can also drive eco-environmental changes by altering land surface processes, in particular land use and land coverage changes. The most serious issue in the TRHR, which poses a threat to the eco-environment, is grassland degradation induced by overgrazing and rodents. As shown in Figure 14a, the theoretical livestock capacity in the TRHR in 2008 ranged from 22.9 × 10^4^ sheep units in Tongde County to 371.2 × 10^4^ sheep units in Zhiduo County, with a mean value of 85.4 × 10^4^ sheep units. The actual livestock capacity ranged from 32.0 × 10^4^ sheep units in Maduo County to 194.0 × 10^4^ sheep units in Xinghai County, with a mean value of 105.6 × 10^4^ sheep units. Livestock overload is very common in the TRHR; the largest livestock overload capacity occurs in Xinghai County at 142.2 × 10^4^ sheep units. Only Dari, Tanggulashan, Zaduo, Maduo, Qumalai and Zhiduo counties show actual livestock capacities that are smaller than their theoretical values. Overgrazing can induce grassland degradation by influencing soil properties [49,50]. By affecting the species composition of the vegetation community and the community coverage and biomass, overgrazing indirectly affects the water cycle and the accumulation of organic matter in the soil. Additionally, livestock trampling, feeding and excrement directly affect the structure and chemical properties of the soil [18,49]. Biomass reduction of the vegetation community can directly affect the absorbance of soil water and nutrients by vegetation, resulting in a reduction in organic matter production and surface litter accumulation. The amount of organic matter returned to the soil would be reduced, loosening the soil structure, decreasing the resistance to erosion and increasing the possibility of soil erosion.

With regard to the rodent population, in 2006, the rodent area in the TRHR (Figure 14b) was highest in Maduo County, with an area of 142.5 × 10^4^ ha; the smallest rodent area was in Banma County at 8.3 × 10^4^ ha. Rodents may cause severe grassland degradation, which poses a serious threat to the eco-environment.

### 5.3. Uncertainties in the Eco-Environmental Impact of Climate Change and the Interaction between Factors

Improvements in the water and heat conditions and the promotion of ecological engineering have improved vegetation growth, and productivity has increased significantly. Although vegetation activity has increased, the soil’s anti-erosion ability had seen no obvious improvements, because the increased rainfall erosivity has led to increased soil erosion. These results are similar to the results given in Shao *et al*. [21]. In terms of water resources, glaciers and snow cover melting due to the rise in temperatures resulted in an increase in water and improved water production ability. However, the increase in streamflow caused by glacier and snow melting is not sustainable, and this negative effect threatens water conservation in the TRHR [44,45,46].

Eco-environmental indicators are closely linked, being characterized by trade-offs and synergies across spatio-temporal scales. Eco-environmental indicators are interrelated because of (1) the effects of common drivers or (2) the interactions among multiple indicators. In our research (Figure 6 and Figure 9a), the sediment concentration and water erosion amounts were strongly correlated, possibly due to common hydrological processes and precipitation drivers. Our research showed positive relationships between the NPP and NDVI for the TRHR in 1982 to 2012 (*p* < 0.001), indicating a synergy between the two indicators.

Some uncertainties exist in our assessment of eco-environmental changes, in particular for soil erosion. The estimation of the water erosion involved the application of the USLE combined with remote sensing. The USLE is based on a statistical relationship established from a large number of plot-scale rainfall-erosion experiments [50,51], involving estimates of rill and inter-rill soil detachments on hill slopes from rainfall, soil and soil cover parameters and management factors [52]. Therefore, the USLE is suitable for estimating the effect of hill slope vegetation rehabilitation on soil erosion. However, this effect may have been overestimated in this research, owing to the omission of local sediment deposition effects.

## 6. Conclusion and Implications

The results of this study are summarized in the following points:

(1) In the TRHR, temperature and precipitation experienced sharp increases during the past 57 years. The precipitation trend changed in 1990 and has since been in a state of high volatility. The rate of the temperature increase, initially observed in 1990, has remained highly volatile and has accelerated. The dramatic rise in winter temperatures is an important reason for the increase in the average annual temperature.

(2) During the past 57 years, annual runoff in the LRB and YARB showed increasing trends, whereas the trend in the YRB declined slightly. Runoff is mainly influenced by rainfall, which is jointly controlled by several monsoon systems. The water temperature anomalies at Tangnaihai station in the YRB and at Batang station in the YARB increased significantly (*p* < 0.001) during 1958 to 2007, driven by air temperature changes.

(3) Owing to warming and wetting trends in the TRHR, which provide sufficient water and heat for vegetation growth, the NPP during 1956 to 2012 and the NDVI during 1982 to 2012 showed significant increasing trends with slopes of 13.53 kg C/(hm^2^ a) (*p* < 0.001) and 0.0025/a (*p* < 0.001), respectively.

(4) During 1956 to 2012, the rainfall erosivity presented a significant increasing trend (*p* < 0.01). The water erosion amount increased in 2000 to 2012, as did the erosion per unit area (500 to 2500 t/km^2^, an increase of about 10%). The wind speed saw a significant declining trend (*p* < 0.05), which caused wind erosion to decline and reduced the frequency and duration of sandstorm events.

(5) During 1956 to 2012, the >0 °C annual cumulative temperature and summer FLH increased significantly, showing slopes of 16.28 m/a and 7.30 °C/a (*p* < 0.001), respectively. The glacier runoff therefore presented an obvious increasing trend, with the highest value appearing in the 2000s. The contributions of the glacier runoff to the total runoff were 6.7% in the LRB, 1.6% in the YRB and 11.7% in the YARB.

Considering the climate variability and vulnerability in the TRHR eco-environment, this region is of great significance for investigating the regional eco-environment under the conditions of climate change. The hydrological cycle in this region has particular importance. Thus, adequate planning is necessary, as is active response and adaptation to the possible effects of future climate change, particularly the effects on water resources, to ensure sustainable development and ecological safety for the TRHR. Moreover, the current ecological restoration is the joint effect of ecological engineering and climate fluctuations, but it is a purely local and temporary restoration, not an overall or a fundamental improvement. In order to secure informed policy-making in the layout, development, utilization, protection and management of natural resources in a reasonable and sustainable way, environment managers should pay particular attention to the eco-environmental changes caused by climate change.

## References

[1] Climate Change 2013, The Physical Science Basis, Summary for Policy makers. https://www.ipcc.ch/pdf/assessment-report/ar5/wg1/WG1AR5_SummaryVolume_FINAL.pdf.

[2] Ji F., Wu Z.H., Huang J.P., Chassignet E.P. (2014). Evolution of land surface air temperature trend. Nat. Clim. Change.

[3] Piao S.L., Ciais P., Huang Y., Shen Z., Peng S., Li J., Zhou L., Liu H., Ma Y., Ding Y. (2010). The impacts of climate change on water resources and agriculture in China. Nature.

[4] Lu Y.H., Zhang L.W., Feng X.M., Zeng Y., Fu B.J., Yao X.L., Li J.R., Wu B.F. (2015). Recent ecological transitions in China: Greening, browning, and influential factors. Sci. Rep..

[5] Shi Y.F., Shen Y.P., Kang E.S., Li D.L., Ding Y.J., Zhang G.W., Hu R.J. (2007). Recent and future climate change in northwest China. Clim. Change.

[6] Wang S., Zhang M., Wang B., Sun M., Li X. (2013). Recent changes in daily extremes of temperature and precipitation over the western Tibetan Plateau, 1973–2011. Quatern. Int..

[7] Xin H., Stone R. (2008). Chinese probe unmasks high-tech adulteration with melamine. Science.

[8] Liu J.Y., Xu X.L., Shao Q.Q. (2008). Grassland degradation in the “Three-River Headwaters” region, Qinghai Province. J. Geogr. Sci..

[9] Li W.H., Zhao X.Q., Zhang X.Z., Shi P.L., Wang X.D., Zhao L. (2013). Change mechanism in main ecosystems and its effect of carbon source/sink function on the Qinghai-Tibetan Plateau. Chin. J. Nat..

[10] Liu X.F., Zhang J.S., Zhu X.F. (2014). Spatiotemporal changes in vegetation coverage and its driving factors in the Three-River Headwaters Region during 2000–2011. J. Geogr. Sci..

[11] Li X., Cheng G.D., Jin H.J., Kang E.S., Che T., Jin R., Wu L.Z., Nan Z.T., Wang J., Shen Y.P. (2008). Cryospheric change in China. Glob. Planet. Change.

[12] Immerzeel W.W., van Beek L.P.H., Bierkens M.F.P. (2010). Climate change will affect the Asian water towers. Science.

[13] Ye B., Yang D., Zhang Z.L., Douglas L. K. (2009). Variation of hydrological regime with permafrost coverage over Lena Basin in Siberia. J. Reophys. Res..

[14] Niu L., Ye B.S., Li J., Sheng Y. (2011). Effect of permafrost degradation on hydrological processes in typical basins with various permafrost coverage in Western China. Sci. China: Ear. Sci..

[15] Luo D.L., Jin H.J., Lin L., He R.X., Yang S.Z., Chang X.L. (2012). Degradation of permafrost and cold-environments on the interior and eastern Qinghai Plateau. J. Glaciol. Geocryol..

[16] Fang Y.P. (2012). Managing the three-rivers headwater region, China: From ecological engineering to social engineering. Ambio.

[17] Tong L.G., Xu X.L., Fu Y. (2014). Wetland changes and their responses to climate change in the “three-river headwaters” region of China since the 1990s. Energies.

[18] Sun W.Y., Shao Q.Q., Liu J.Y., Xiao T. (2011). The variation characteristics of soil organic carbon of typical alpine slope grasslands and its influencing factors in the “Three-River Headwaters” Region. J. Nat. Resour..

[19] Bing L.F., Shao Q.Q., liu J.Y., Zhao Z.P. (2011). Runoff characteristic in flood and dry seasons in source regions of Changjiang River and Huanghe River based on wavelet analysis. Sci. Geogra. Sin..

[20] Zhang Y.Y., Zhang S.F., Zhai X.Y., Xia J. (2012). Runoff variation and its response to climate change in the Three Rivers Source Region. J.Geogra. Sci..

[21] Shao Q.Q., Liu J.Y., Fan J.W., Huang L., Fan J.W., Xu X.L., Wang J.B. (2013). Integrated assessment on the effectiveness of ecological conservation in Sanjiangyuan National Nature Reserve. Geogr. Res..

[22] Remote Sensing Monitoring Center of Eco-environment in Qinghai Province, China. http://www.sanjiangyuan.org.cn/.

[23] The National Geomatics Center of China. http://www.ngcc.cn/.

[24] U.S. Geological Survey. http://www.usgs.gov/.

[25] National Meteorological Data Sharing Service System. http://data.cma.gov.cn/.

[26] National Climate Center. http://cmdp.ncc-cma.net/Monitoring/monsoon.php.

[27] Cold and Arid Regions Science Data Center. http://westdc.westgis.ac.cn/.

[28] Gan T.Y. (1998). Hydroclimatic trends and possible climatic warming in the Canadian Prairies. Water Resour. Res..

[29] Mu X.M., Zhang X.Q., Gao P., Wang F. (2010). Theory of double curves and its applications in hydrology and meteorology. J. China Hydrol..

[30] Merriam C.F. (1937). A comprehensive study of the rainfall on the Susquehanna Valley. Trans. Am. Geophys. Union.

[31] Searcy J.K., Hardisoni C.H., Langbein W.B. Double Mass Curves with a Section Fitting Curves to Cyclic Data Water Supply Paper 1541-B. https://pubs.er.usgs.gov/publication/wsp1541B.

[32] Allen R.G., Pereira L.S., Raes D., Martin S. Crop Evapotranspiration–Guidelines for Computing Crop Water Requirements—FAO Irrigation and Drainage Paper 56. https://appgeodb.nancy.inra.fr/biljou/pdf/Allen_FAO1998.pdf.

[33] Lieth H. (1975). Modelling the primary productivity of the world. Primary Productivity of the Biosphere.

[34] Braithwaite R.J. (1995). Positive degree-day factor for ablation on the Greenland ice sheet studied by energy-balance modelling. J. Glaciol..

[35] Zhang Y., Liu S.Y., Ding Y.J. (2006). Spatial Variation of Degree-day Factors on the Observed Glaciers in Western China. J. Geogr. Sci..

[36] Braithwaite R.J., Olesen O.B. (1993). Seasonal variation of ice ablation at the margin of the Greenland ice sheet and its sensitivity to climate change, Qamanarssup sermia, West Greenland. J Glaciol..

[37] Liu S.Y., Ding Y.J., Li J., Shangguan D.H., Zhang Y. (2006). Glaciers in response to recent climate warming in western China. Quat. Sci..

[38] Liu S.Y., Ding Y.J., Ye B.S. (1996). Study on the mass balance of the Glacier No.1 at the headwaters of the Urumqi River using degree-day method. Proceeding of the Fifth Chinese Conference on Glaciology and Geocryology.

[39] Gao X., Ye B.S., Zhang S.Q., Qiao C.J., Zhang X.W. (2010). Glacier runoff variation and its influence on river runoff during 1961–2006 in the Tarim River Basin, China. Sci. China Earth Sci..

[40] Williams J.R., Arnold J.G. (1997). A system of erosion-sediment yield models. Soil Technol..

[41] Zhang W.B., Fu J.S. (2002). Rain erositivity estimation under different rainfall amount. Resour. Sci..

[42] Liu B.Y., Zhang K.L., Xie Y. (2002). An Empirical Soil Loss Equation. Proceeding of 12th ISCO.

[43] Fryrear D.W., Bilbro J.D., Saleh A., Schomberg H., Stout J.E., Zobeck T.M. (2000). RWEQ: Improved wind erosion technology. J. Soil Water Conserv..

[44] Kang E.S., Cheng G.D., Dong Z.C. (2002). Glacier-Snow Water Resources and Mountain Runoff in the Arid Area of Northwest China.

[45] Kang E.S., Shen Y.P., Li X. (2004). Assessment of the Glacier and Snow Water Resources in China.

[46] Yang Z.N. (1991). Glacier Water Resources in China.

[47] Xie Z.C., Wang X., Kang E.S., Feng Q.H., Li Q.Y., Cheng L. (2006). Glacial runoff in China: An evaluation and prediction for the future 50 years. J. Glaciol. Geocryol..

[48] Cao L.G., Pan S.M. (2014). Changes in precipitation extremes over the “Three-River Headwaters” region, hinterland of the Tibetan Plateau, during 1960–2012. Quat. Int..

[49] Shao J. A., Shao Q. Q., and liu J. Y. (2009). Soil property and its formation of ecosystems in three river sources, Qinghai. Geogr. Res..

[50] Ciesiolka C.A.A., Yu B., Rose C.W., Ghadiri H., Lang D. (2006). Improvement in soil loss estimation in USLE type experiments. J. Soil Water Conserv..

[51] Kinnell P.I.A. (2008). Discussion: Misrepresentation of the USLE in “Is sediment delivery a fallacy?”. Earth Surf. Proc. Land.

[52] Tattari S., Barlund I. (2001). The concept of sensitivity in sediment yield modelling. Phys.Chem. Earth PT. B.

